# Fe_3_O_4_@iron-based metal–organic framework nanocomposite [Fe_3_O_4_@MOF (Fe) NC] as a recyclable magnetic nano-organocatalyst for the environment-friendly synthesis of pyrano[2,3-d]pyrimidine derivatives

**DOI:** 10.3389/fchem.2023.1193080

**Published:** 2023-05-15

**Authors:** Ghader Hootifard, Enayatollah Sheikhhosseini, Sayed Ali Ahmadi, Mahdieh Yahyazadehfar

**Affiliations:** Department of Chemistry, Kerman Branch, Islamic Azad University, Kerman, Iran

**Keywords:** magnetic nanocatalyst, metal–organic frameworks, pyrano[2,3-d]pyrimidines, 2,6-pyridinedicarboxylic acid, green chemistry, microwave irradiation

## Abstract

Various pyrano[2,3-d]pyrimidines were synthesized by the multicomponent reaction of aldehydes, malononitrile, and acidic C–H compounds such as barbituric acid through the tandem Knoevenagel–Michael cyclocondensation pathway in an environmentally friendly reactive medium in the presence of a recoverable nanocomposite. This nanocomposite includes Fe_3_O_4_ nanoparticles placed on an organometallic framework. The synthesized Fe_3_O_4_@iron-based metal–organic framework nanocomposite was characterized using scanning electron microscopy, energy dispersive X-ray spectroscopy, X-ray powder diffraction, a vibrating sample magnetometer, and thermogravimetric analysis.

## 1 Introduction

Catalysis is one of the fundamental and beneficial processes in the chemical industries as they can reduce reagent-based wastes and enhance the selectivity of reactions in standard chemical transformations, hence minimizing potential by-products. Nanocatalysts are distinguished from their bulk counterparts due to their significantly smaller size with a unique shape which can significantly increase the surface area-to-volume ratio (Sajjadifar et al., 2019).

As a novel class of porous materials, metal–organic frameworks (MOFs) have recently attracted a great deal of attention due to their well-defined structure and high surface area. Made of an inorganic metal cluster and polyfunctional organic linkers, MOFs have found diverse applications in optics, gas storage, sensors, gas separation, biomedicine, energy technologies, and catalysis (Zeraati et al., 2021).

Among various kinds of MOFs, iron-based MOFs are developed as heterogeneous catalysts for organic reactions due to their high dispersibility, structural efficiency, and abundant catalytically active sites (Bai et al., 2016; Rimoldi et al., 2017). Given the strong affinity of Fe^3+^ ions toward carboxylate groups of organic linkers, Fe-based MOFs have highly stable chemical structures, which can facilitate recycling and increase the chance of microwave-assisted synthesis. Moreover, the low toxicity, redox activity, and cost-effectiveness of Fe^3+^ further added to the popularity of MOF (Fe) (Doan et al., 2016; Nguyen et al., 2016).

Thanks to their reusability and facile separation, Fe_3_O_4_ magnetic nanoparticles (Fe_3_O_4_ MNPs) have found wide applications in heterogeneous catalysis (Liu et al., 2015). Numerous functional materials have been studied with the Fe_3_O_4_ MNPs as a core. However, the easy oxidation by other substances and strong magnetic properties lead to aggregation and instability of Fe_3_O_4_ MNPs (Chen et al., 2019). Hence, some functional materials (e.g., MOF, carbon, polymer, PEG, and metal oxide) are loaded on the surface of the Fe_3_O_4_ MNPs to increase the stability and dispersion (Kim et al., 2013; Mirfakhraei et al., 2018; Moradi et al., 2018; Bakhteeva et al., 2019).

Chemists are interested in synthesizing compounds with a minimum of one pyran nucleus, as revealed by the current synthesis of various pyran derivatives such as pyranopyran, pyranochromene, and pyranopyrimidine (Raj et al., 2010; Solhy et al., 2010; Boominathan et al., 2011; Brahmachari and Banerjee, 2014).

Pyrano[2,3-d]pyrimidines are important heterocyclic compounds with remarkable pharmaceutical and biological effects, such as antibacterial, antitumor, antioxidant, anticancer, antifungal, anti-hypertension (Daneshvar et al., 2018), analgesics, and herbicidal (Amirnejat et al., 2020) features. The three-component condensation of aromatic aldehydes, barbituric acid, and malononitrile is among the simplest and most popular strategies in the synthesis of pyrano[2,3-d]pyrimidine dione-derived materials. So far, various catalysts have been introduced for the synthesis of pyrano[2,3-d]pyrimidine compounds, including (NH_4_)_2_HPO_4_, (Balalaie et al., 2008), SBA-Pr-SO_3_H (Ziarani et al., 2013), TMU-16-NH_2_ (Beheshti et al., 2018), KF (Elinson et al., 2014), ZnFe_2_O_4_ (Khazaei et al., 2015), the Fe_3_O_4_@SiO_2_@(CH_2_)_3_-Urea-SO_3_H/HCl magnetic nanoparticle (Zolfigol et al., 2016), Et_3_N (Azarifar et al., 2012), and ZnO nano powder (Maleki et al., 2016). Some studies have also reported the synthesis of pyrano[2,3–d]pyrimidines in the presence of ionic liquids (Karami et al., 2019), Bronsted acids (Mahmoudi et al., 2019), and magnetic catalysts (Sajjadifar and Gheisarzadeh, 2019). Most of these catalysts have had considerable drawbacks, including long reaction time, harsh reaction conditions, difficulties in the recovery of the catalyst, high-cost reagents, low yield, laborious workup, use of dangerous organic solvents, costly and moisture-sensitive reagents, and application of disposable dangerous catalysts. Therefore, research has mainly focused on the development of environmentally friendly and recyclable catalysts capable of effective synthesis of pyrano[2,3-d]pyrimidine derivatives.

**SCHEME 1:**
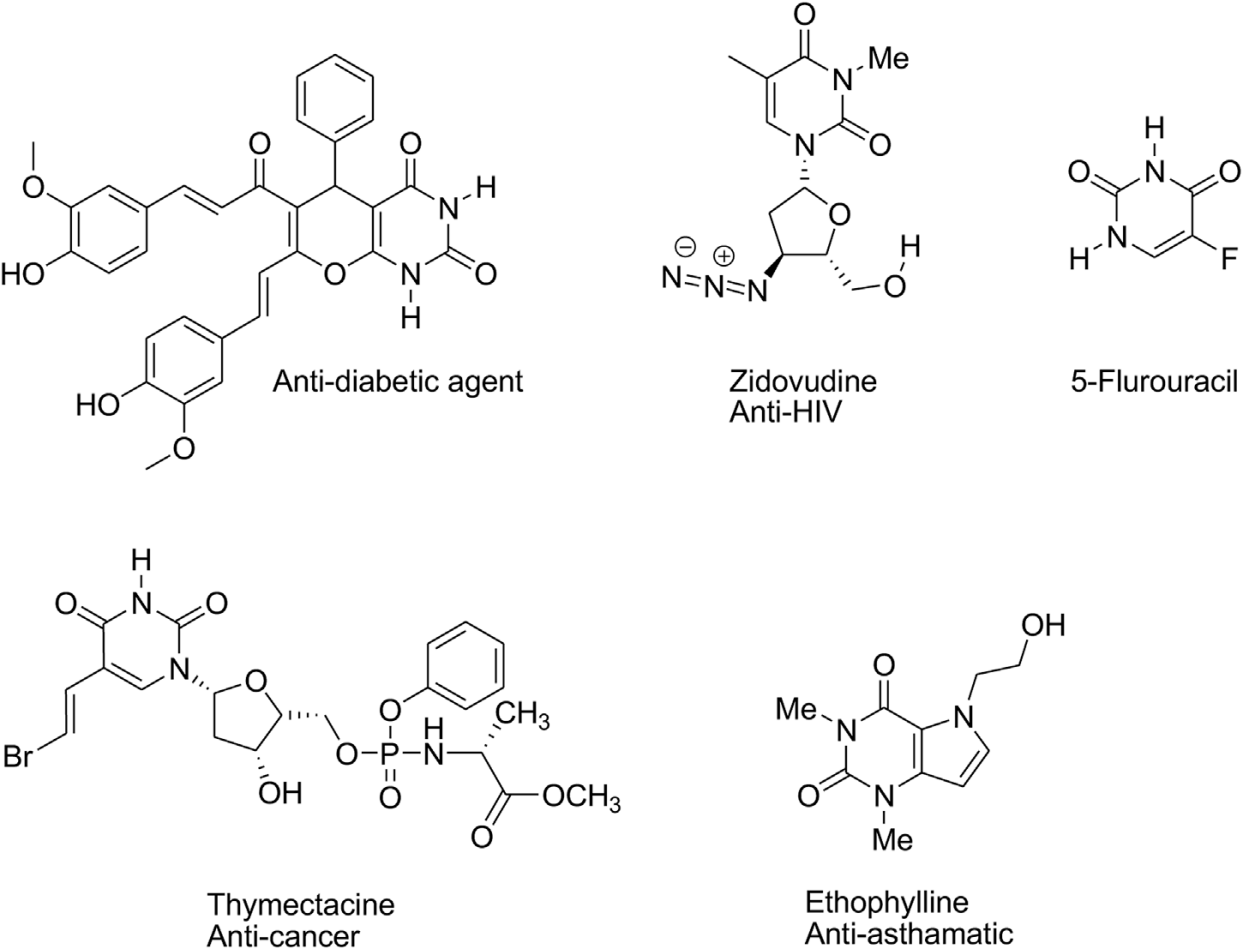
Some bioactive pyrimidine-annulated heterocyclic compounds.

Scheme 1 presents some examples of bioactive pyrimidine-annulated heterocyclic compounds with various medicinal properties.

In continuation of our attempts to the synthesis of heterocyclic compounds via MCR methodologies (Yahyazadehfar et al., 2019; Moghaddam-Manesh et al., 2020a; Moghaddam-Manesh et al., 2020b; Moghaddam-Manesh et al., 2020c; Yahyazadehfar et al., 2020), this research is focused on the synthesis of an eco-friendly and recyclable Fe_3_O_4_@iron-based metal–organic framework nanocomposite [Fe_3_O_4_@MOF (Fe) NC] using microwave irradiation to synthesize pyrano[2,3-d]pyrimidine scaffolds through three-component reactions of aryl aldehyde derivatives, malononitrile with barbituric acid. While the magnetic characteristics of the catalysts facilitate their quick and straightforward solid-phase separation from solution, further functionality can be introduced by magnetic nanoparticles through the synthesis of magnetic framework composites. This study reports a facile preparation of a magnetic metal–organic framework (Fe_3_O_4_@MOF (Fe)) as a new and cost-effective catalyst by microwave irradiation and use in the synthesis of pyrano[2,3-d]pyrimidine heterocycles.

## 2 Experimental section

### 2.1 Chemicals and reagents

Iron (III) nitrate, iron (II) chloride, ammonia, and 2,6-pyridinedicarboxylic acid were provided by Sigma-Aldrich. All the reagents and solvents were purchased from Merck chemical company and used without further purification.

### 2.2 Material characterization

Products were characterized in terms of their physical constants in comparison to authentic samples based on FT-IR spectroscopy. The purity of the substrates and reaction progress were determined by thin-layer chromatography (TLC) on aluminum-backed plates coated with Merck Kieselgel 60 F254 silicagel. Melting points were measured using the Electrothermal 9,100 Apparatus in open capillary tubes. IR spectra were recorded on a JASCO FT-IR-4000 spectrophotometer device in the range of 400–4,000 cm^−1^. ^1^H and ^13^C NMR spectra were also attained using a Bruker AC (400 MHz for ^1^H NMR and 100 MHz and ^13^C NMR) and DMSO-d_6_ as solvents. A Philips analytical PC-APD X-ray diffractometer operating with Kα (α_2_, λ_2_=1.54439 Å) and graphite mono-chromatic Cu (α_1_, λ_1_=1.54056 Å) radiations was used for X-ray powder diffraction (XRD) to explore the structure of the product. Then, scanning electron microscopy (SEM) and energy-dispersive X-ray (EDX) spectroscopy (KYKY & EM 3200) were applied to observe CB Fe-MOF@Fe_3_O_4_ NFC. Magnetization measurements were also carried out with a Lake Shore vibrating sample magnetometer (model 7407) under magnetic fields at room temperature.

### 2.3 Synthesis of a nano-organocatalyst

#### 2.3.1 Synthesis of magnetic Fe_3_O_4_ nanoparticles

First, 16 mmol (4.325 g) of iron (III) chloride was dissolved in a minimum amount of deionized water. In another beaker, 8 mmol (1.590 g) of iron (II) chloride was dissolved in a minimum volume of deionized water. The two solutions were mixed followed by the dropwise addition of 10 mL of 25% ammonia which immediately resulted in the formation of a large amount of black Fe_3_O_4_ precipitate. Stirring was continued for 20 min. Finally, the products were magnetically separated, rinsed with distilled water four times, and dried in an oven at 80°C.

#### 2.3.2 Synthesis of MOF (Fe)

To achieve a solution containing 13.365 mmol (2.233 g) of 2,6-pyridinedicarboxylic acid-linker in deionized water at 80°C, 4.455 mmol (1.8 g) iron nitrate was dissolved in the minimum amount of deionized water and stirred at 80°C. After overnight refrigeration, the resulting Fe-MOF precipitates were collected. To remove the raw materials, the resulting products were washed three times with boiling water and dried at 70°C for 12 h.

#### 2.3.3 Synthesis of an Fe_3_O_4_@MOF (Fe) nanocomposite

Dried Fe-MOF (0.6 g; 1.050 mmol) was dispersed in deionized water. Next, 0.137 g (0.35 mmol) of Fe_3_O_4_ was added, and the mixture was stirred at 80°C for 10 min to achieve a homogeneous solution. The final powder mixture was transferred to a glassy vial for microwave irradiation. Then, the glassy vial was immediately inserted in the microwave oven (145 W) and irradiated for 1 h. The raw materials were eliminated from the resulting products through washing with acetic acid. Ultimately, the dried powder was calcined at 170°C.

### 2.4 General procedure for the preparation of pyrano[2,3-d]pyrimidine derivatives

A mixture of aryl aldehyde derivatives (1 mmol), malononitrile (1 mmol), barbituric acid (1 mmol), Fe_3_O_4_@MOF (Fe) NC (25 w %), and 10 mL H_2_O/EtOH (1:1) was heated at 90°C for an appropriate time duration. After completion of the reaction (monitored by TLC (n-hexane/EtOAc, 70:30), the reaction mixture was cooled down, and 5 mL hot EtOH was added. Since the catalyst is insoluble in ethanol, the magnetic nanocatalyst was separated from the reaction vessel using an external magnet bar. After evaporation of EtOH, the expected products were formed and washed with hot H_2_O.

### 2.5 Selected spectral data

7-Amino-5-(4-chlorophenyl)-2,4-dioxo-1,3,4,5-tetrahydro-2H-pyrano[2,3-d]pyrimidine-6-carbonitrile (**4a**): Yield: 95%. M.p. = 226°C–229°C. IR (KBr, cm^−1^), 3,315, 3,187, 2,198, 1718, 1,637. ^1^H NMR (DMSO-*d*_*6*_, 400 MHz): *δ* = 4.23 (s, 1H, benzylic CH), 7.13–7.34 (m, 6H, 2H-NH_2_, 4H-Ar), 11.06 (s, 1H, NH), 12.07 (s, 1H, NH).

7-Amino-2,4-dioxo-5-(3,4,5-trimethoxyphenyl)-1,3,4,5-tetrahydro-2H-pyrano[2,3-d]pyrimidine-6-carbonitrile (**4b**): Yield: 98%. M.p. = 249°C–252°C. IR (KBr, cm^−1^), 3,452, 3,288, 2,197, 1705, 1,663.^1^H NMR (DMSO-*d*_*6*_, 400 MHz): *δ* = 3.71 (s, 3H, OCH_3_), 3.80 (s, 6H, OCH_3_), 4.42 (s, 1H, benzylic CH), 6.44 (s, 2H, H-Ar), 7.35 (s, 2H, NH_2_), 10.40 (s, 1H, NH), 11.10 (s, 1H, NH). 7-amino-5-(2-methoxyphenyl)-2,4-dioxo-1,3,4,5-tetrahydro-2H-pyrano[2,3-d]pyrimidine-6-carbonitrile (**4c**): Yield: 98%. M.p. = 209°C–212°C. IR (KBr, cm-1), 3,365, 3,304, 2,175, 1721, 1,674. ^1^H NMR (DMSO-*d*_*6*_, 400 MHz): *δ* = 3.71 (s, 3H, OCH3), 4.46 (s, 1H, benzylic CH), 6.83–6.92 (m, 2H, H-Ar), 7.01 (brs, 2H, NH2), 7.04–7.19 (m, 2H, H-Ar), 10.96 (s, 1H, NH), 11.96 (s, 1H, NH). 7-amino-5-(2-hydroxyphenyl)-2,4-dioxo-1,3,4,5-tetrahydro-2H-pyrano[2,3-d]pyrimidine-6-carbonitrile (4d): Yield: 91%. M.p. = 171°C–172°C. IR (KBr, cm^−1^), 3,445, 2,201, 1726, 1,643. ^1^H NMR (DMSO-*d*_*6*_, 400 MHz): *δ* = 4.89 (S, 1H, benzylic CH), 6.66 (s, 2H, NH_2_), 7.05–7.40 (m, 4H, H-Ar), 9.73 (s, 1H, OH), 10.98 (s, 1H, NH), 11.02 (s, 1H, NH). 7-amino-5-(2,4-dichlorophenyl)-2,4-dioxo-1,3,4,5-tetrahydro-2H-pyrano[2,3-d]pyrimidine-6-carbonitrile (**4e**): Yield: 93%. M.p. = 239°C–242°C. IR (KBr, cm^−1^), 3,390, 3,327, 2,195, 1717, 1,678. ^1^H NMR (DMSO-*d*_*6*_, 400 MHz): *δ* = 4.71 (s, 1H, benzylic CH), 7.18 (s, 2H, NH_2_), 7.32–7.51 (m, 3H, H-Ar), 11.06 (s, 1H, NH), 12.10 (s, 1H, NH).

7-Amino-5-(3-nitrophenyl)-2,4-dioxo-1,3,4,5-tetrahydro-2H-pyrano[2,3-d]pyrimidine-6-carbonitrile (**4f**): Yield: 97%. M.p. = 264°C–266°C. IR (KBr, cm^−1^), 3,417, 3,203, 2,192, 1711, 1,659. ^1^HNMR (400 MHz, DMSO-*d*_*6*_): *δ* = 4.47 (s, 1H, benzylic CH), 7.30 (br s, 2H, NH_2_), 7.61 (t, J = 8.0 Hz, 1H, H-Ar), 7.75 (d, J = 7.8 Hz, 1H, H-Ar), 8.0.6 (t, J = 2 Hz, 1H), 8.08–8.12 (m, 2H, H-Ar), 11.12 (s, 2H, NH), 12.18 (s, 1H, NH).

7-Amino-5-(2-nitrophenyl)-2,4-dioxo-1,3,4,5-tetrahydro-2H-pyrano[2,3-d]pyrimidine-6-carbonitrile **(4g)**: Yield: 94%. M.p. = 229°C–233°C. IR (KBr, cm-1), 3,468, 3,365, 2,198, 1705, 1,659. ^1^HNMR (400 MHz, DMSO-d6): δ = 5.00 (s, 1H, benzylic CH), 7.27 (brs, 2H, NH2), 7.44–7.82 (m, 4H, H-Ar), 11.04 (s, 1H, NH), 12.12 (s, 1H, NH).

7-Amino-5-(4-nitrophenyl)-2,4-dioxo-1,3,4,5-tetrahydro-2H-pyrano[2,3-d]pyrimidine-6-carbonitrile (**4h**): Yield: 97%. M.p. = 240°C–243°C. IR (KBr, cm^−1^): 3,373, 3,186, 2,198, 1720, 1,637. ^1^HNMR (400 MHz, DMSO-*d*_*6*_): *δ* = 4.40 (s, 1H, benzylic CH), 7.24 (brs, 2H, NH_2_), 7.51 (d, *J* = 8.25 Hz, 2H, H-Ar), 8.14 (d, *J* = 8 Hz, 2H, H-Ar), 11.09 (s, 1H, NH), 12.15 (s, 1H, NH). 7-amino-2,4-dioxo-5-(p-tolyl)-2,3,4,5-tetrahydro-1H-pyrano[2,3-d]pyrimidine-6-carbonitrile (**4i**): Yield: 96%. M.p. = 226°C–228°C. IR (KBr, cm^−1^): 3,391, 3,321, 2,199, 1716, 1,677. ^1^HNMR (400 MHz, DMSO-*d*_*6*_): *δ* = 2.23 (s, 3H, CH_3_), 4.14 (s, 1H, benzylic CH), 7.06 (brs, 6H, 2H-NH_2_, 4H-Ar), 11.03 (s, 1H, NH), 12.02 (s, 1H, NH). 7-amino-2,4-dioxo-5-(m-tolyl)-1,3,4,5-tetrahydro-2H-pyrano[2,3-d]pyrimidine-6-carbonitrile (**4j**): Yield: 91%. M.p. = 230°C–233°C. IR (KBr, cm^−1^): 3,415, 3,319, 2,194, 1711, 1,661. ^1^HNMR (400 MHz, DMSO-*d*_*6*_): *δ* = 2.25 (s, 3H, CH_3_), 4.15 (s, 1H, benzylic CH), 6.97–7.15 (m, 6H, 2H-NH_2_, 4H-Ar), 11.03 (s, 1H, NH), 12.04 (brs, 1H, NH).

7-Amino-2,4-dioxo-5-phenyl-1,3,4,5-tetrahydro-2H-pyrano[2,3-d]pyrimidine-6-carbonitrile (**4k**): Yield: 95%. M.p. = 206°C–208°C. IR (KBr, cm^-1^): 3,385, 3,335, 2,194, 1714, 1,677. ^1^HNMR (400 MHz, DMSO-*d*_*6*_): *δ* = 4.20 (s, 1H, benzylic CH), 7.08–7.25 (m, 7H, 2H-NH_2_, 5H-Ar), 11.04 (s, 1H, NH), 12.05 (brs, 1H, NH).

7-Amino-5-(4-bromophenyl)-2,3,4,5-tetrahydro-2,4-dioxo-1H-pyrano[2,3-d]pyrimidine-6-carbonitrile (**4l**): Yield 97%, m.*p* = 228°C–232°C. IR (KBr, cm^−1^): 3,207, 3,153, 3,091, 2,195, 1,693, 1,678. ^1^H NMR (300 MHz, DMSO-*d*_*6*_ *,* ppm) δ: 4.21 (s, 1H, CH), 7.16 (d, 4H, *J* = 8.25, 2H-Ar, 2H-NH_2_), 7.45 (d, 2H, *J* = 7.75, H-Ar), 11.04 (s, 1H, NH), 12.06 (brs, 1H, NH); ^13^C NMR (75 Hz, DMSO-*d*_6_): δ = 162.9, 158.0, 152.8, 149.9, 132.7, 132.6, 131.5, 130.1, 120.2, 119.5, 88.4, 58.7, 35.7.

## 3 Results and discussion

### 3.1 Characterization of the Fe_3_O_4_@MOF (Fe) NC

**FIGURE 1:**
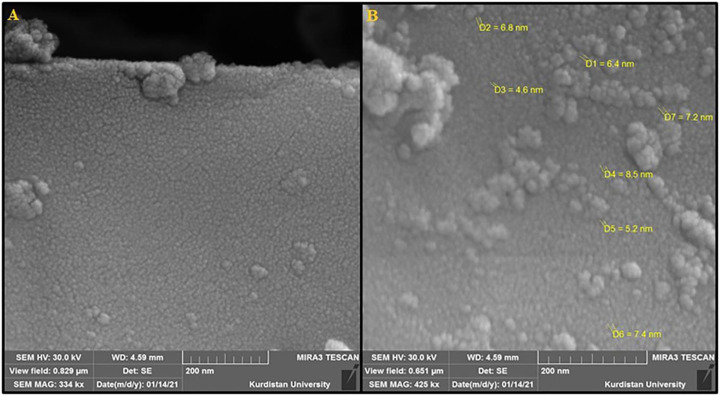
**(A)** FE-SEM image and **(B)** high-resolution FE-SEM image of the Fe_3_O_4_@MOF (Fe) NC.

The size and morphology of the Fe_3_O_4_@MOF (Fe) NC were characterized by SEM (Figure 1). The SEM image represents the spherical morphology of the Fe_3_O_4_@MOF (Fe) NC. The as-synthesized Fe_3_O_4_@MOF (Fe) NC showed uniform size and shape with relative mono-dispersion. The particles have a narrow size distribution with less than 10 nm.

**FIGURE 2:**
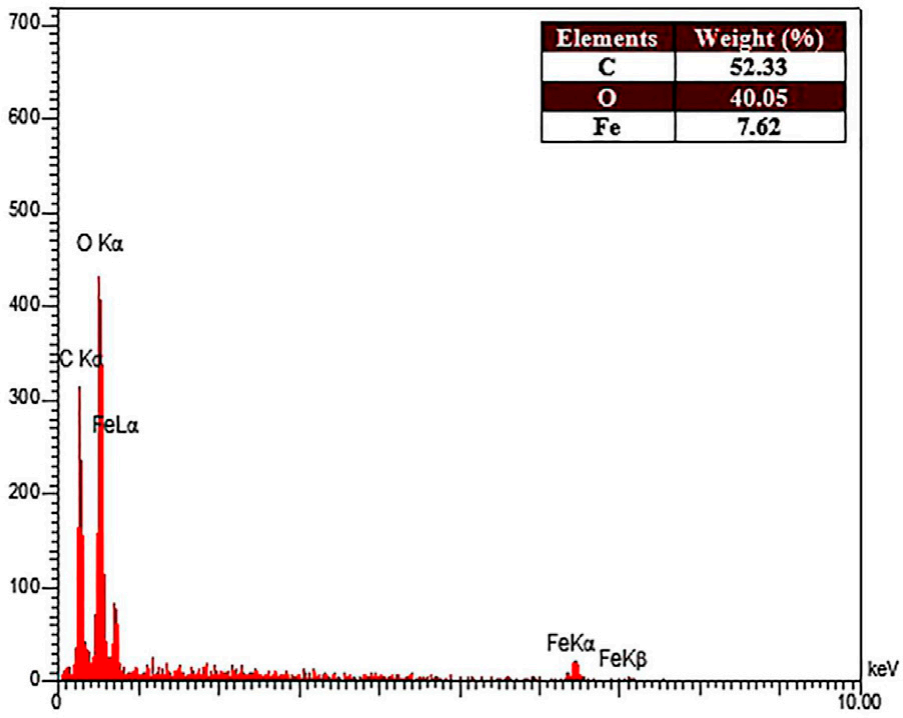
EDX spectra of the Fe_3_O_4_@MOF (Fe) NC.

Moreover, the purity of the synthesized Fe_3_O_4_@MOF (Fe) NCs was studied after calcination EDX (Figure 2). The EDX results revealed that the prepared Fe_3_O_4_@MOF (Fe) NC consisted of carbon, oxygen, and iron (52.33, 40.05, and 7.62 w/w%, respectively) (inset of Figure 2) with no impurity, indicating that the composite sample is composed of Fe_3_O_4_ and MOF (Fe).

**FIGURE 3:**
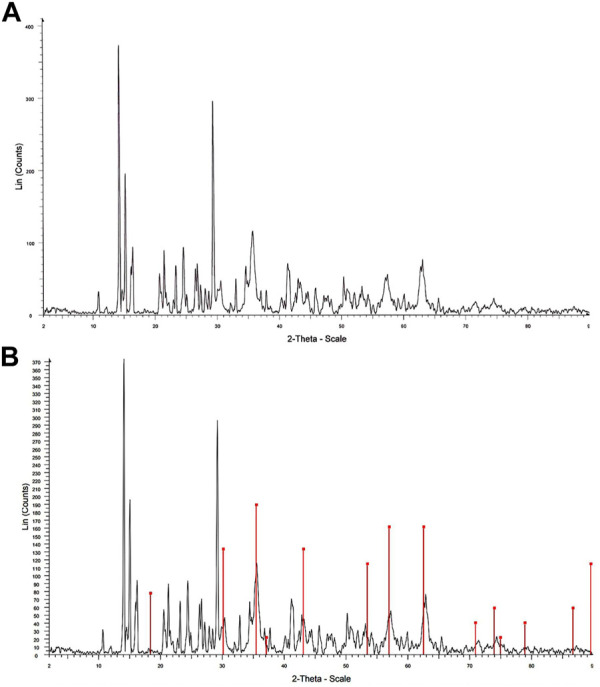
XRD pattern of the **(A)** Fe_3_O_4_@MOF (Fe) NC and **(B)** Fe_3_O_4_@MOF (Fe) NC simulated on phase (Fe_3_O_4_).

The XRD patterns of the Fe_3_O_4_@MOF (Fe) NC and Fe_3_O_4_@MOF (Fe) NC simulated on phase (Fe_3_O_4_) are, respectively, shown in Figures 3A, B. In both XRD patterns, the peaks are recorded in the 2θ range of 10°–70°. The nanocomposite showed six characteristic diffraction peaks at 2θ = 30.56°, 35.83°, 43.08°, 53.30°, 57.47°, and 62.74°, which can be indexed to the (220), (311), (400), (422), (511), and (440) crystalline planes of the cubic inverse spinel structure of Fe_3_O_4_ (JCPDS no. 88–0315). Therefore, the crystalline structure of Fe_3_O_4_ MNPs was not notably damaged by MOF (Fe) coating (Ali et al., 2019). Moreover, Figure 3B shows the XRD patterns of the Fe_3_O_4_@MOF (Fe) NC simulated on phase (Fe_3_O_4_). According to this figure, the XRD pattern showed multiple diffraction peaks that were assigned to MOF (Fe) as a poly-crystalline structure. As an important result, the Fe_3_O_4_@MOF (Fe) NC was successfully simulated on phase (Fe_3_O_4_). Using the Debye–Scherrer equation, D=Kλ/*β*cosθ, the average crystallite size (D) was estimated by measuring the width of the Bragg reflections, where λ stands for the wavelength of X-ray (1.54056 Å for Cu lamp), K represents the Scherrer constant (0.9), θ shows half of the Bragg diffraction angle, and *β* denotes half of the width of the maximum intensity diffraction peak. The average crystallite size of the Fe_3_O_4_@MOF (Fe) NC was 10 nm, indicating the excellent dispersity and crystal structures of the Fe_3_O_4_@MOF (Fe) NC with no agglomeration.

**FIGURE 4:**
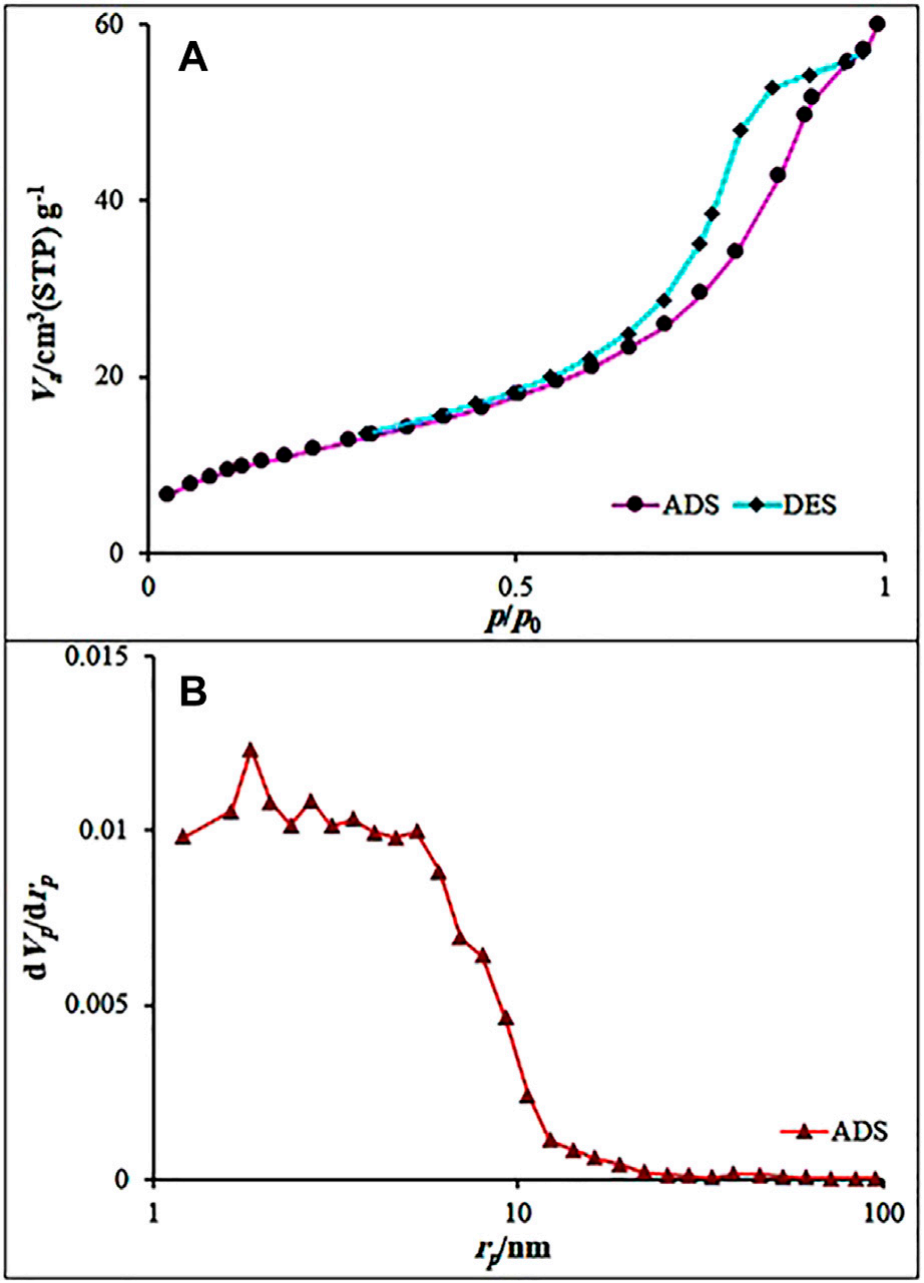
**(A)** N_2_ adsorption–desorption isotherms of the Fe_3_O_4_@MOF (Fe) NC and **(B)** BJH results obtained for the Fe_3_O_4_@MOF (Fe) NC.

Nitrogen adsorption–desorption isotherms were recorded at 77 K. The Fe_3_O_4_@MOF (Fe) NC was analyzed using the BET and 2D-NLDFT methods (Figure 4). A slow absorption was observed in the P/P_0_ range of 0.0–0.2, followed by a rapid increase in the P/P_0_ range of 0.2–1.0. These results suggest IV isotherm with a type H2 hysteresis loop for the Fe_3_O_4_@MOF (Fe) NC samples, which is a typical feature of materials with uniform mesoporous and inkbottle shape pores. The BJH pore volume (VBJH) and the BET surface area (SBET) of the samples were 0.093 cm^3^/g and 113.93 m^2^/g, respectively. These results revealed the wide pore openings and a high porosity of Fe_3_O_4_@MOF (Fe) NC, making it one of the best catalysts in the organic transformations.

**FIGURE 5:**
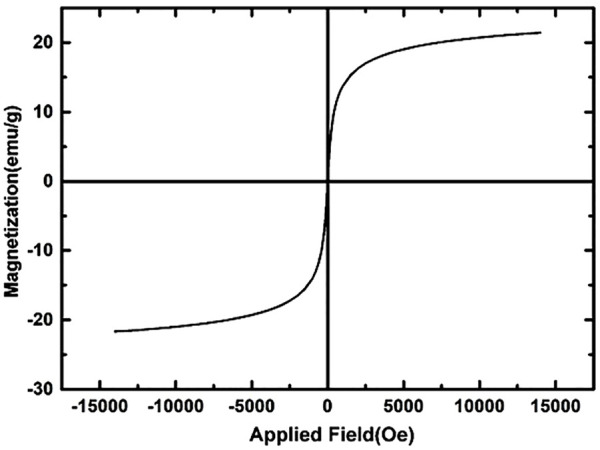
VSM magnetization curves of the Fe_3_O_4_@MOF (Fe) NC.

The magnetic hysteresis loop of the Fe_3_O_4_@MOF (Fe) NC in the presence of magnetic field was measured using a vibrating sample magnetometer (VSM). Figure 5 shows the hysteresis loops of the Fe_3_O_4_@MOF (Fe) NC at room temperature. This figure proved the super-paramagnetic properties of Fe_3_O_4_@MOF (Fe) NC. The Fe_3_O_4_@MOF (Fe) NC exhibited small remanent magnetization (M_r_, 1.98 emu/g) and coercivity (H_c_, 0.9 Oe), indicating its suitable magnetic behavior and saturation magnetization (M_s_, 21.2 emu/g). The M_s_ value of 21.2 emu/g suffices for magnetic separation by a conventional magnet. Therefore, the Fe_3_O_4_@MOF (Fe) NC can be used as a recoverable catalyst in the organic transformations.

**FIGURE 6:**
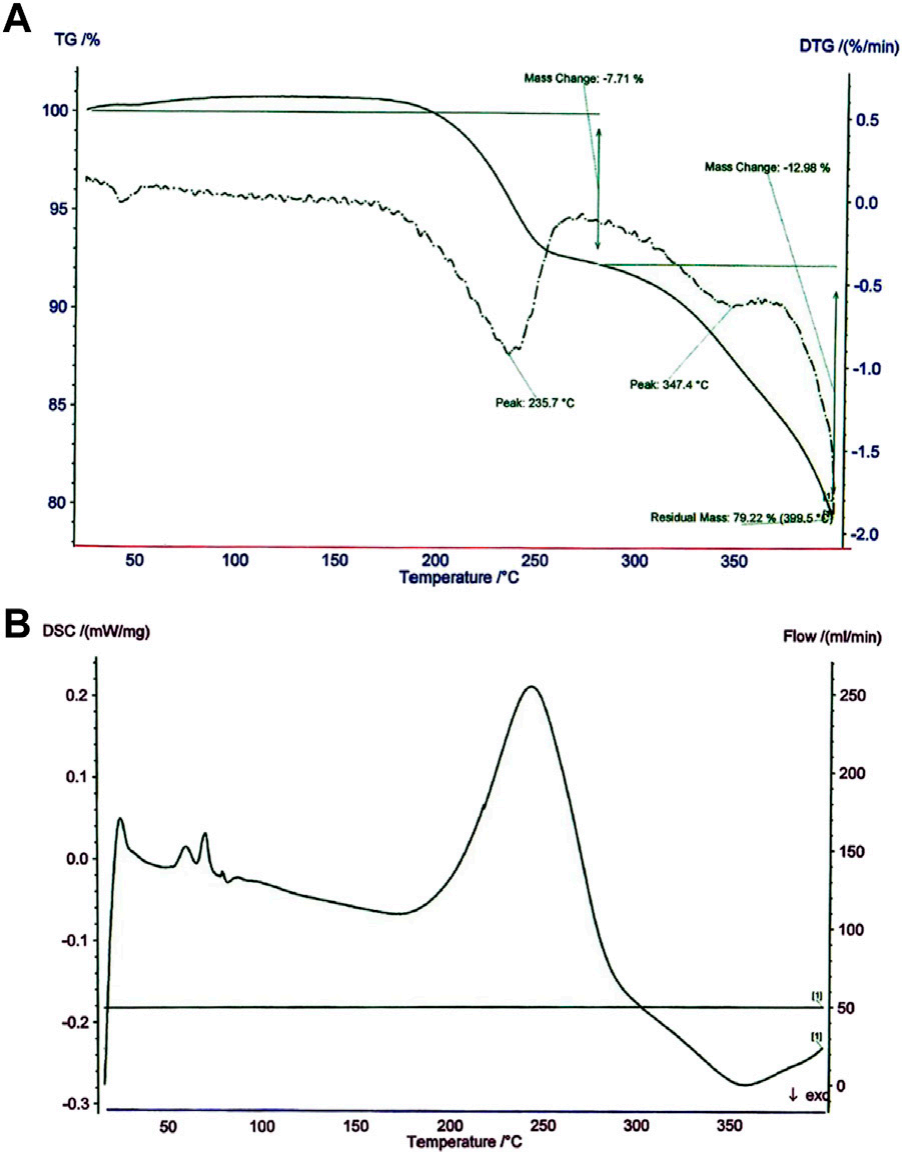
**(A)** TG and **(B)** DSC curves of the Fe_3_O_4_@MOF (Fe) NC.

Thermogravimetric analysis (TGA) and DSC were utilized to assess the thermal behavior of the Fe_3_O_4_@MOF (Fe) NC (Figures 6A, B). Therefore, TGA was applied on an STA-1500 thermoanalyzer under the inert condition in the scope 30°C–350°C and at a heating rate of 10°C min^−1^. Thermal analysis of the Fe_3_O_4_@MOF (Fe) NC as a final product is presented in Table 1. The results reveal appropriate thermal stability without weight loss. A partial diminish in the weight (1%) was recorded at a temperature from 50°C to 100°C. This is due to the removal of trapped solvents such as the water in the pores and on the MOF (Fe) skeleton. Proportionate to the temperature ascent from 100°C to 235°C, a 7.71% reduction in weight was observed. That probably corresponds to decomposing linker on the skeleton. The weight loss of dissociation of coordinated water (12.98%) for the catalyst was estimated in the range of 235°C–347°C. The main weight loss of 79.22% of the catalyst is observed in the range of 347°C–399°C, which is connected with the final decomposition. Therefore, obtained data show high thermal stability in elevated temperatures.

**TABLE 1:** TGA and DSC data of Fe_3_O_4_@MOF (Fe) NC.

Step no.	Temperature (°C)	Result
I	47	Vanished solvent
II	235	Ligand decomposition
III	347	Dissociation of coordinated water
IV	399	Final decomposition

**FIGURE 7:**
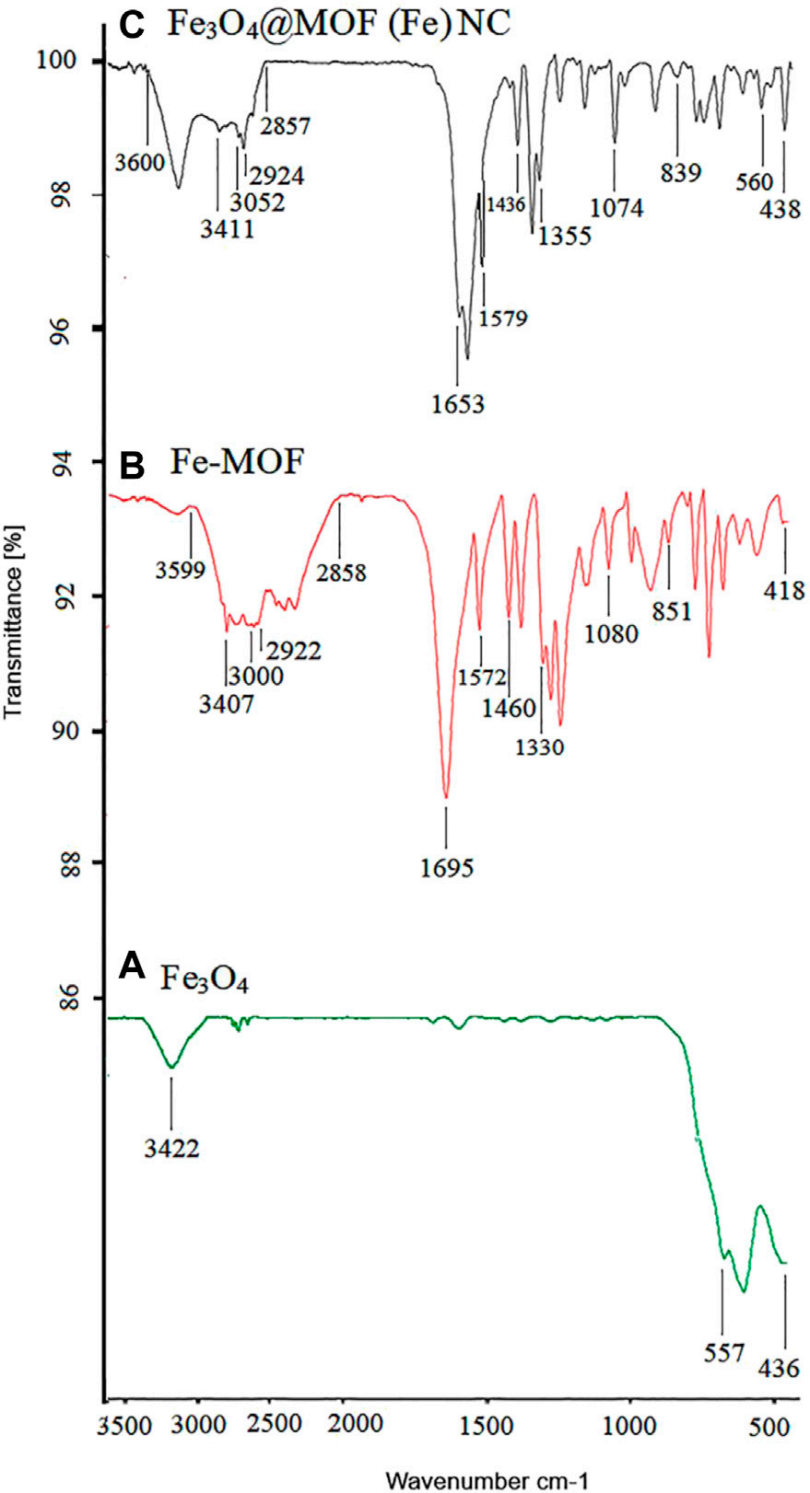
IR (KBr, υ/cm^-1^) curve of **(A)** Fe_3_O_4_, **(B)** synthesized Fe-MOF, and **(C)** the Fe_3_O_4_@MOF (Fe) NC.

The FT-IR spectra of Fe_3_O_4_, Fe-MOF, and the Fe_3_O_4_@MOF (Fe) NC are shown in Figure 7. The presence of nanoparticles in the complex structure of the Fe_3_O_4_@MOF (Fe) NC is confirmed by emergence of some peaks at 436, 557, and 438, 560 cm^−1^ corresponding to Fe–O vibration in Fe_3_O_4_ and the Fe_3_O_4_@MOF (Fe) NC, respectively. The stretching frequencies of hydroxyl groups on the surface of the nanoparticles appeared as a peak at 3,422 cm^-1^ whose broadening and low intensity can be due to intermolecular hydrogen bonding and the chelation of iron atom with the oxygen atom of Fe_3_O_4_, respectively. Moreover, two bands at 3,052 and 2,924 cm^−1^ can be attributed to C–H stretching, while the two peaks at 1,436 and 1,579 cm^−1^ are related to C–C and C–N bonds of pyridine, respectively. The band at 1,355 cm^−1^ can be also ascribed to the NO^−3^ counter ion. The bands at 1,074 and 839 cm^−1^, respectively, confirm the presence of C–N groups and Fe–N bond, whereas the band at 438 cm^−1^ indicates the presence of an Fe–O bond. Vibration frequencies indicate the presence of Fe-MOF in the structure of the Fe_3_O_4_@MOF (Fe) NC. The absorption of acidic OH in the nano-organocatalyst at 2,857 to 3,600 cm^−1^ is related to the acidic OH of the ligand, which is seen in the region of 2,858–3,599 cm^−1^ in Fe-MOF. The adsorption of the CO ligand in Fe-MOF appeared at 1,695 cm^−1^, while this adsorption in nano-organocatalyst emerged at 1,653 cm^−1^. Other absorptions at 1,074, 839, and 438 cm^-1^ in nano-organocatalyst are, respectively, equivalent to the absorptions at 1,080 and 851 cm^−1^ in Fe-MOF and absorption of 436 cm^−1^ in nanoparticles, indicating the presence of magnetite nanoparticles on the structure of Fe-MOF and formation of the complex Fe_3_O_4_@MOF (Fe) NC.

### 3.2 Synthesis of pyrano[2,3-d]pyrimidine via the nano-organocatalyst Fe_3_O_4_@MOF (Fe) NC

**SCHEME 2:**
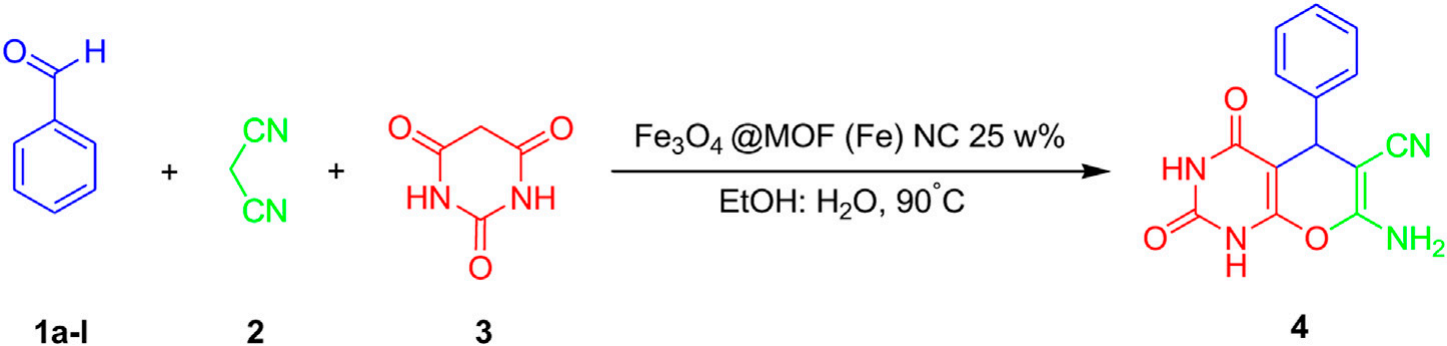
Synthesis of pyrano[2,3-d]pyrimidines.

The Fe_3_O_4_@MOF (Fe) NCs were designed, synthesized, and characterized before their employment in the one-pot synthesis of pyrano[2,3-d]pyrimidine derivatives using a variety of aldehydes, malononitrile, and barbituric acid (Scheme 2).

Initially, the model reaction had to be formed by selecting the 4-chlorobenzaldehyde, malononitrile, and barbituric acid mixture (containing 1 mmol of each), followed by meticulous investigation of the impact of different factors, including the catalyst amount, solvent, and temperature to obtain the most desirable reaction conditions.

Accordingly, the impacts of different solvents, temperatures, and catalyst amounts were examined on the model reaction of 4-chlorobenzaldehyde, malononitrile, and barbituric acid to optimize the reaction conditions and evaluate the primary influential parameters on the catalyzed pyrano[2,3-d]pyrimidine reactions. Lower yields and longer reaction times were reported in the absence of the solvent but the presence of 20 w% catalyst at 90°C, suggesting the effects of the solvent on the reaction advancement. The EtOH: H_2_O ratio of 1:1 represents the most effective solvent for this reaction, as it accelerated the reaction more than other solvents and solvent-free conditions (Table 2). The use of ethanol: water mixture as a green solvent can offer several advantages including the absence of carcinogenic complications, eco-friendliness, relatively lower operating costs, and clean reaction conditions. The impact of the organic–aqueous EtOH interface and stabilization of the reaction intermediate can be described by the effective establishment of hydrogen bonding. A solvent with considerable polarity and faster heat transfer can offer optimum conditions for intermediate generation.

**TABLE 2:** Optimization of the reaction conditions for the synthesis of pyrano[2,3-d]pyrimidine derivatives using the CB Fe-MOF@Fe_3_O_4_ NFC.

Entry	Catalyst	Solvent	Tem (^0^C) (°C)	Time (min/h)	Yield (%)
1	Fe_3_O_4_@MOF (Fe) NC 20%	CHCl_3_	90	17 min	76
2	Fe_3_O_4_@MOF (Fe) NC 20%	CH_3_CN	90	25 min	61
3	Fe_3_O_4_@MOF (Fe) NC 20%	CH_2_Cl_2_	90	17 min	76
4	Fe_3_O_4_@MOF (Fe) NC 20%	EtOH	90	4 min	94
5	Fe_3_O_4_@MOF (Fe) NC 20%	MeOH	90	7 min	88
6	Fe_3_O_4_@MOF (Fe) NC 20%	MeOH: H_2_O	90	5 min	90
7	Fe_3_O_4_@MOF (Fe) NC 20%	Solvent-free	90	55 min	68
8	Fe_3_O_4_@MOF (Fe) NC 20%	EtOH: H_2_O	90	5 min	93
9	Fe_3_O_4_@MOF (Fe) NC 20%	EtOH: H_2_O	25	40 min	43
10	Fe_3_O_4_@MOF (Fe) NC 20%	EtOH: H_2_O	120	5 min	93
11	Fe_3_O_4_@MOF (Fe) NC 5%	EtOH: H_2_O	90	10 min	77
12	Fe_3_O_4_@MOF (Fe) NC 10%	EtOH: H_2_O	90	8 min	80
13	Fe_3_O_4_@MOF (Fe) NC 15%	EtOH: H_2_O	90	7 min	85
14	Fe_3_O_4_@MOF (Fe) NC 25%	EtOH: H_2_O	90	2 min	98
15	Fe_3_O_4_@MOF (Fe) NC 30%	EtOH: H_2_O	90	2 min	98
16	Fe_3_O_4_@MOF (Fe) NC 35%	EtOH: H_2_O	90	2 min	98
17	Catalyst-free	EtOH: H_2_O	90	9 h	Trace
18	Fe_3_O_4_	EtOH: H_2_O	90	50 min	25

Reaction conditions: 3-chlorobenzaldehyde (1 mmol), malononitrile (1 mmol), barbituric acid (1 mmol), and Fe_3_O_4_@MOF (Fe) NC, under different conditions.

The effect of reaction temperature was also examined to improve the yields and interaction conditions (Table 2, entries 8, 9). A temperature decline from 90°C to 25°C enhanced the reaction time while decreasing the reaction rate and the product yield (Table 2, entry 9). On the other hand, an increment in the reaction temperature from 90 to 120°C led to no further improvements in the yield or decrease in the reaction time at different temperatures (Table 2, entry10).

The effect of catalyst loading was examined after optimization of the effects of solvent and temperature. In the absence of the catalyst, only a trace of product was observed at 90°C within 9 h (Table 2, entry 17), indicating the essential role of the catalyst in this transformation. A decrease in the catalyst loading from 20 w% to 5 w% reduced the yield of the relative product (Table 2, entries 8, 11–13). In the catalyst loading of 20–35 w%, the catalyst content of 25% led to the highest efficiency, below which no effects were detected on the yield or reaction improvement (Table 2, entries 8, 14–16). Thus, 25 w% of the Fe_3_O_4_@MOF (Fe) NC as the nano-organocatalyst in H_2_O/EtOH (1:1) at 90°C resulted in the most desirable reaction conditions (Table 2, entry 14).

The effect of the reaction scope on the model reaction was investigated following the optimization of the reaction conditions. Based on the findings (Table 3), the electronic impact of various substitutions on the aromatic aldehydes had no effects on the product yield. Activation of the reactions was totally similar in both (electron-donating/withdrawing) substitutions at the ortho/meta/para positions on the aromatic aldehyde (Table 3).

**TABLE 3:** Preparation of pyrano[2,3-d]pyrimidine using the Fe_3_O_4_@MOF (Fe) NC as the nano-organocatalyst.

Entry	R (aldehyde)	Product	Time (min)	Yield (%)^b^	$\frac{m.p.\;\left(℃\right)}{Found\;Reported\;\left[ref.\right]}$
1	4-ClC_6_H_4_-	4a	5	95	226-229 228-230 Maleki et al. (2017)
2	3,4,5-(OCH_3_)_3_C_6_H_2_-	4b	20	98	249-252 247-249 Asadpour Behzadi et al. (2020)
3	2-CH_3_OC_6_H_4_-	4c	5	98	209-212 209-210 Khazaei et al. (2015)
4	2-OHC_6_H_4-_	4d	7	91	171-172 169-170 Mohamadpour (2022)
6	2,4-Cl_2_C_6_H_3_-	4e	7	93	239-242 238-240 Maleki et al. (2019)
7	3-NO_2_C_6_H_4_-	4f	3	97	264-266 266-268 Maleki et al. (2019)
8	2-NO_2_C_6_H_4_-	4g	4	94	229-233 228-230 Maleki et al. (2019)
9	4-NO_2_C_6_H_4_-	4h	2	97	240-243 239-240 Maleki et al. (2017)
10	4-CH_3_C_6_H_4_-	4i	5	96	226-228 223-225 Asadpour Behzadi et al. (2020)
11	3-CH_3_C_6_H_4_-	4j	6	91	230-233 228-229 Sheikhhosseini et al. (2016)
12	C_6_H_5_-	4k	2	95	206-208 206-210 Maleki et al. (2017)
13	4-BrC_6_H_4_-	4l	4	97	228-232 230-231 Maleki et al. (2017)

^a^ Reaction conditions: aldehyde (1 mmol), malononitrile (1 mmol), and barbituric acid (1 mmol) in the presence of the Fe_3_O_4_@MOF (Fe) NC (25 w %) in 10 mL H_2_O/EtOH (1:1) was heated at 90°C for appropriate time.

^b^ Isolated yields.

**SCHEME 3:**
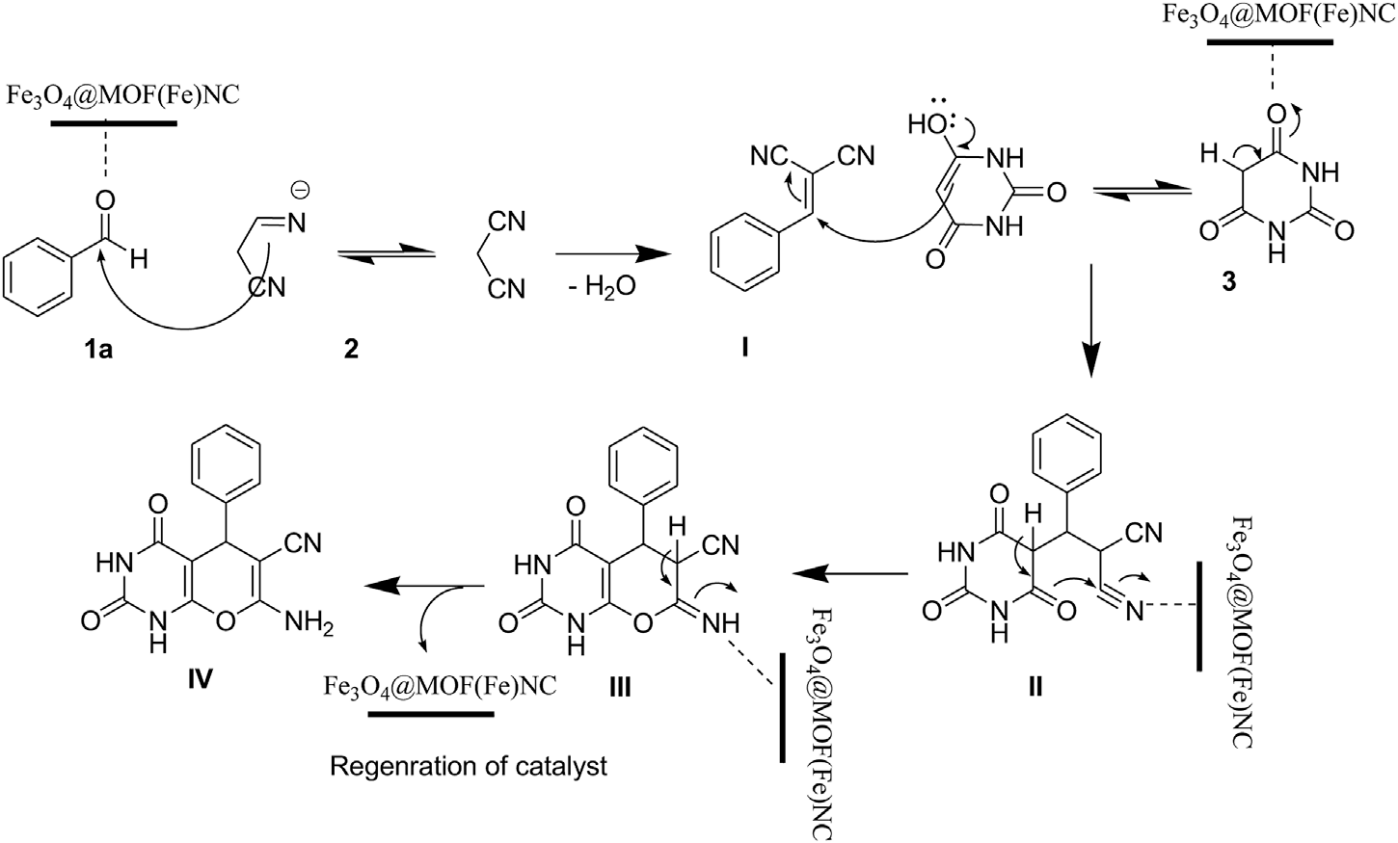
A possible mechanism for the preparation of pyrano[2,3-d]pyrimidinone derivatives.

To justify this mechanism, Knoevenagel condensation and then cyclization resulting in xanthene derivatives are ascribed to the special role of the Fe_3_O_4_@MOF (Fe) NC as a catalyst (Scheme 3). As the Fe_3_O_4_@MOF (Fe) NC is a Lewis acidic catalyst, benzaldehyde was first partially bound with the Fe_3_O_4_@MOF (Fe) NC for the carbonyl carbon activation, after which C=C bonds were formed with the active methylene group of malononitrile **2** for the formation of intermediate 2-benzylidenemalononitrile (**I**). The next step involved the formation of C–C bonds of intermediates (**I**) which had activated barbituric acid for the intermediate (**II**) formation. Then, the intramolecular cyclization of intermediates (**II**) gave intermediates (**III**), followed by the NH group protonation which yielded the eventual product (**4**) and regenerated the catalyst.

**FIGURE 8:**
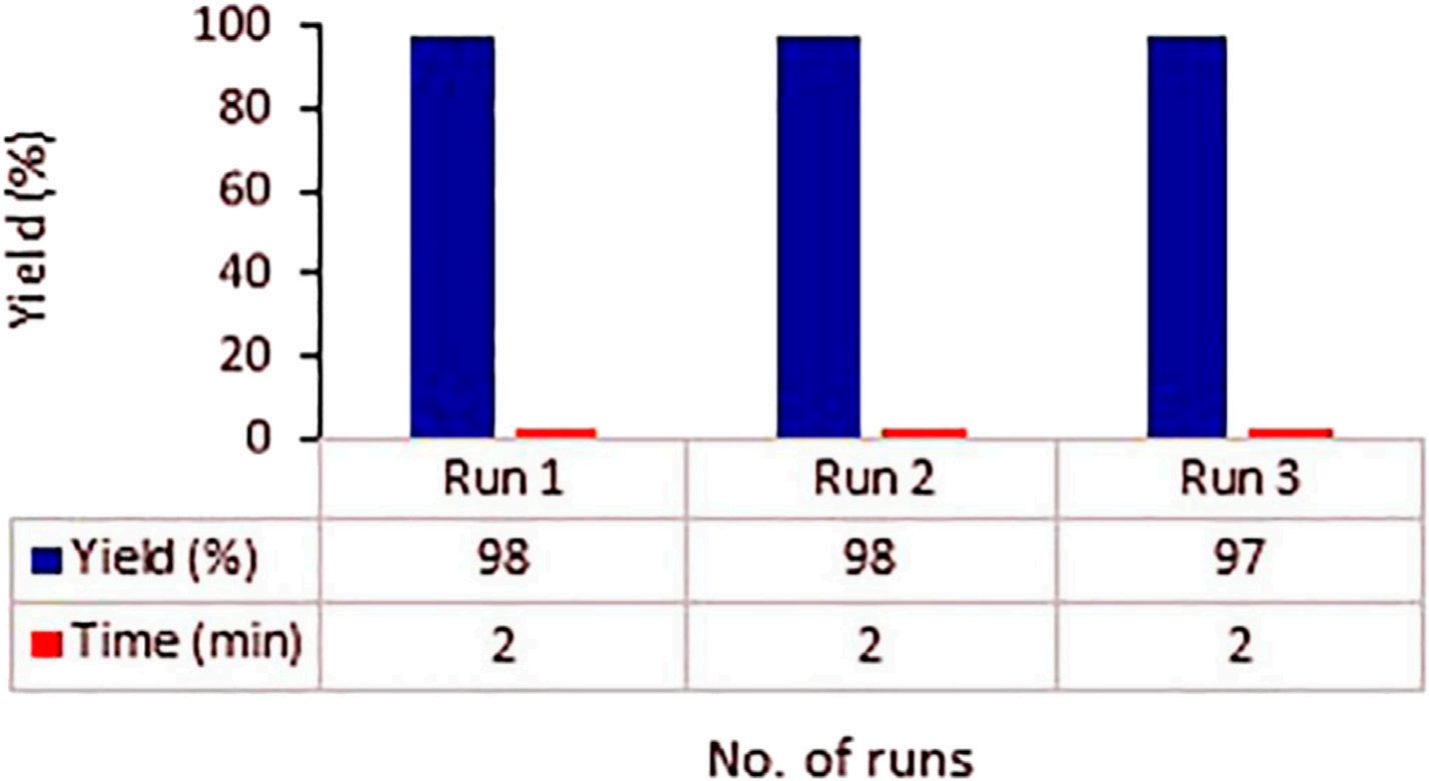
Reusability of the catalyst at reflux.

Recovery and recyclability of the catalyst can significantly contribute to different industries, commercial areas, and green chemistry. The most important advantages of the reported magnetic nanocomposite are its reusability and recovery due to its magnetic properties. The reusability of the prepared catalyst was checked in a model reaction for the pyrano[2,3-d]pyrimidine derivative preparation under optimized conditions for at least three runs. For this purpose and reaction completion, the nanocatalyst was simply separated by an external magnetic field followed by washing with ethanol, drying in an oven at 40°C. After three runs, no considerable decrease was detected in the catalytic activity of the samples (Figure 8).

**FIGURE 9:**
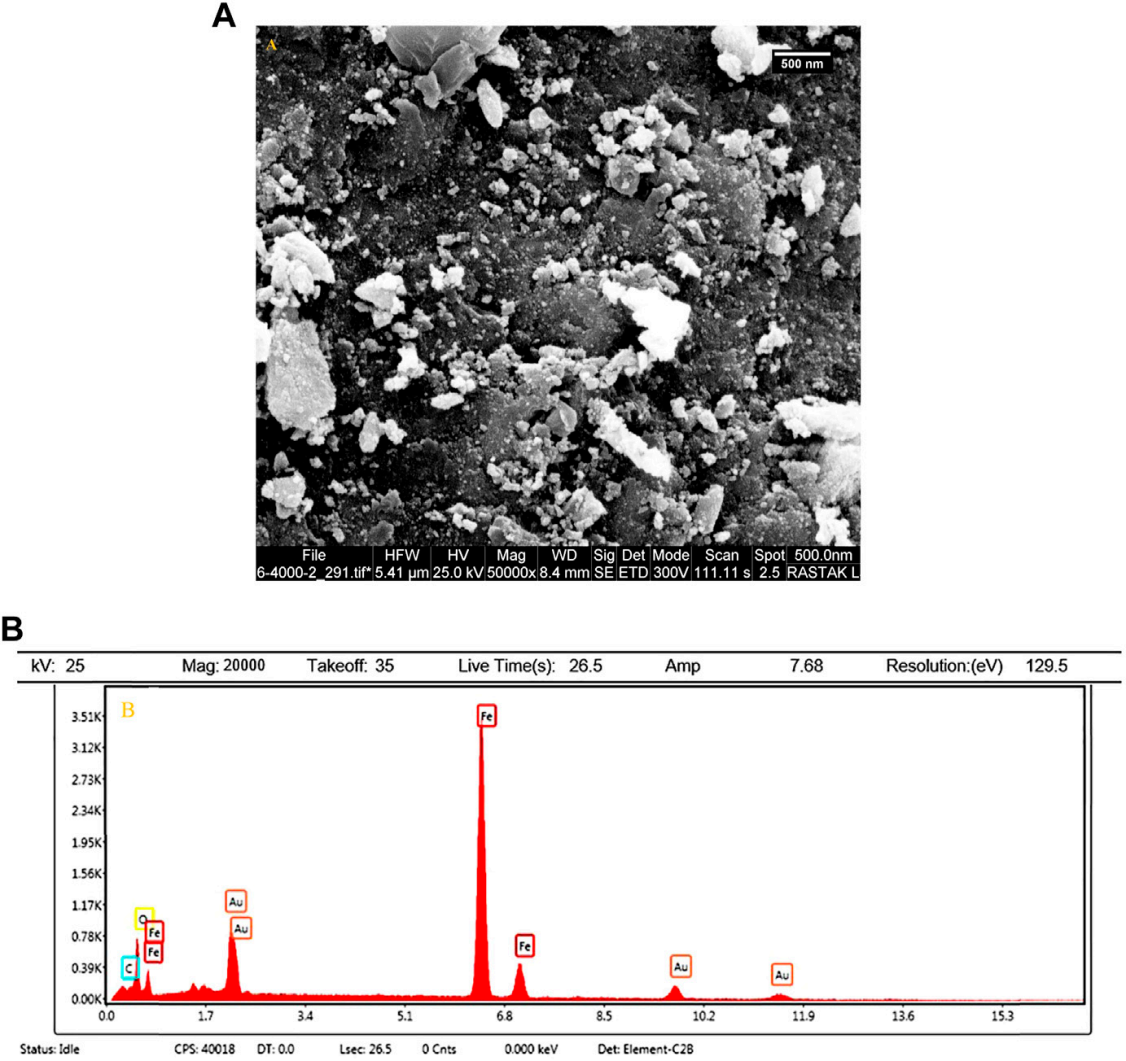
**(A)** SEM image and **(B)** EDX analysis of the Fe_3_O_4_@MOF (Fe) NC after the catalytic procedure.

The SEM image and EDX elemental analysis of the Fe_3_O_4_@MOF (Fe) NC after the catalytic procedure are shown in Figures 9A, B. Based on Figure 9A, the particle size distribution of samples is uniform, and there is no evidence of agglomeration in the structure. As an important result, the Fe_3_O_4_@MOF (Fe) NC is stable in terms of morphology and surface. Also, the EDX elemental analysis confirmed the presence of related elements (Fe, O, and C) of the Fe_3_O_4_@MOF (Fe) NC in the final product after the catalytic procedure (Figure 9B).

Table 4 compares the catalytic potential of several previously reported catalytic reactions with the Fe_3_O_4_@MOF (Fe) NC for the synthesis of pyrano[2,3-d]pyrimidine scaffolds. According to the current paper, the Fe_3_O_4_@MOF (Fe) NC has extraordinary potential as an economic and easily accessible catalyst to mildly and conveniently synthesize these biologically active heterocyclic compounds at a considerable yield and short reaction time. The results revealed that the Fe_3_O_4_@MOF (Fe) NC is an excellent nano-organocatalyst in terms of both reaction time and product yield.

**TABLE 4:** Comparison of the synthesis of pyrano[2,3-d]pyrimidines in the presence of the Fe_3_O_4_@MOF (Fe) NC with other catalysts reported in the literature.

Entry	Catalyst	Amount of catalyst	Conditions	Time (min)	Yield (%)	Ref.
1	[TPPHSP]Br	2 mol%	H_2_O:EtOH, reflux	70	80	Karami et al. (2019)
2	L-proline	5 mol%	H_2_O:EtOH, r.t	30–90	68–86	Asadpour Behzadi et al. (2020)
3	CoFe_2_O_4_@glutamin-Dy	40 mg	H_2_O/r.t./UV	15	92	Daraie et al. (2019)
4	γ-Fe_2_O_3_@SiO_2_@[Bis APTES]Cl_2_-NPs	10 mg	H_2_O:EtOH (2:1)/80 C	12	85	Sorkhabi et al. (2021)
5	ZnO@CuO	30 mg	H_2_O, reflux	12	91	Sorkhabi et al. (2021)
6	CoFe_2_O_4_@FA-Er	0.025 g	H_2_O, 100 °C	15	96	Sorkhabi et al. (2021)
7	CoFe_2_O_4_@FA-Er	30 mg	Sonication/H_2_O/80°C	10	92	Sorkhabi et al. (2021)
8	Zn [(l) proline]_2_	20 mol%	EtOH, reflux	30–720	80–92	Sajjadifar and Gheisarzadeh (2019)
9	SBA-Pr-SO_3_H	20 mol%	Solvent-free, 140°C	10–45	30–91	Sajjadifar and Gheisarzadeh (2019)
10	α-Fe_2_O_3_	10 wt%	EtOH, rt	30	93	Sorkhabi et al. (2021)
11	Nano-Fe_3_O_4_@APTES@isatin-SO_3_H MNPs	20 mg	EtOH:H_2_O, reflux	7–25	86–95	Sajjadifar and Gheisarzadeh (2019)
12	Mefenamic acid	0.5 g	Ethanol, reflux	25	97	Asadpour Behzadi et al. (2020)
13	Iron ore pellet	1 number	H_2_O:EtOH, reflux	8–31	73–93	Daraie et al. (2019)
14	Fe_3_O_4_@MOF (Fe) NC	0.25 w%	H_2_O:EtOH, 90 C	2	97	This work

## 4 Conclusion

In summary, a novel and efficient magnetic Fe_3_O_4_@MOF (Fe) NC was synthesized as a nano-organocatalyst by microwave irradiation. The nanocatalyst was fully characterized by various techniques, including FE-SEM, EDX, XRD, TGA, BET, and VSM. The catalytic activity of the Fe_3_O_4_@MOF (Fe) NC as a solid Lewis acid magnetic nanocatalyst was approved in the preparation of pyrano[2,3-d]pyrimidine scaffolds through the tandem Knoevenagel–Michael cyclocondensation reactions in aqueous/aqueous ethanol media at 90°C. This nanocatalyst can be easily recovered by an appropriate external magnet and reused at least three times with no considerable decline in its catalytic effects. High catalytic activity and easy magnetic separation from the reaction medium are two significant factors in the performance of the Fe_3_O_4_@MOF (Fe) NC in the organic transformations. This novel protocol offers advantages such as short reaction times, eco-friendly catalyst, recyclability, no need for column chromatography, easy workup, and progress of the reaction under green conditions.

## Data Availability

The original contributions presented in the study are included in the article/Supplementary Materials; further inquiries can be directed to the corresponding author.
